# Prevalence and Relevance of Pruritus in Pregnancy

**DOI:** 10.1155/2017/4238139

**Published:** 2017-09-25

**Authors:** Justyna Szczęch, Artur Wiatrowski, Lidia Hirnle, Adam Reich

**Affiliations:** ^1^Department of Dermatology, Venereology and Allergology, Wroclaw Medical University, Wroclaw, Poland; ^2^Department of Dermatology, University of Rzeszów, Rzeszów, Poland; ^3^1st Department and Clinic of Gynecology and Obstetrics, Wroclaw Medical University, Wroclaw, Poland; ^4^2nd Department and Clinic of Gynecology and Obstetrics, Wroclaw Medical University, Wroclaw, Poland

## Abstract

Pregnant women are at greater risk to suffer from chronic pruritus, but data on this symptom in this group is very limited. The aim of this study was to investigate the prevalence, clinical characteristics, and the importance of pruritus in pregnant women. A total of 292 consecutive pregnant women at the 33.0 ± 6.1 weeks of gestation (WoG) were recruited into this prospective, cross-sectional study. All patients underwent thorough anamnesis and detailed physical examination with the special emphasis on pruritus. Pruritus was assessed according to Visual Analogue Scale (VAS). Quality of life was measured with the Dermatology Life Quality Index (DLQI). The point prevalence of pruritus was 20.2% (*n* = 59), while pruritus prevalence during the entire pregnancy was 38.0% (*n* = 111). Pruritus started on average at the 27.2 ± 7.6 WoG; it was significantly more common among women in third trimester. The mean VAS was 4.8 (±2.4) points. The DLQI scoring significantly correlated with VAS (*r* = 0.52, *p* < 0.001). Based on the results of our study about one-third of women suffer from pruritus during pregnancy. Many of them find it a very distressing and disturbing symptom.

## 1. Introduction

Data on pruritus in pregnancy is rather limited, and physicians treating pregnant women may underestimate its frequency and clinical meaningfulness. Most published papers concerning this symptom during pregnancy focused mainly on itch occurring in intrahepatic cholestasis of pregnancy (ICP) and other pregnancy-specific dermatoses, leaving the problem of idiopathic itch in pregnant women without proper investigation.

Pregnancy is a state that leads to various hormonal, metabolic, and immunologic changes, which may influence the functioning and structure of the skin and mucous membranes. Almost 90% of the pregnant women will present with the signs of hyperpigmentation, mainly visible in physiologically highly pigmented areas, for example, genitals, perineum, periumbilical skin, and areolae [1, 2]. Equally often, on the abdomen may occur the striae gravidarum, or “stretch marks,” which are the result of skin stretching combined with genetic and hormonal changes [1, 3]. In nearly 75% of pregnancy cases physicians will observe gray-brown patches located on the face, previously termed as “mask of pregnancy,” namely, melasma [1]. Besides the above described skin changes pregnant women also present with some physiological hair, nail, and vascular changes, which need to be differentiated from pathological symptoms to avoid unnecessary treatment [1]. Moreover, there is a group of specific dermatoses of pregnancy, in which we can distinguish atopic eruption of pregnancy (AEP), polymorphic eruption of pregnancy (PEP), pemphigoid gestationis (PG), and ICP [4]. The endocrinology of pregnancy involves increased activity of maternal adrenal and pituitary glands, along with physiological development of fetal endocrine glands. Progesterone and estrogen, among other hormones (e.g., increased cortisone levels), are major factors influencing skin during pregnancy [5]. It is possible that these changes may alter the pruritus pathway and contribute to itch in susceptible individuals [6].

In 2007, the International Forum for the Study of Itch (IFSI) established a new classification of chronic itch which allows physicians to assign all patients with pruritus to one of three groups including subjects with pruritus on diseased (inflamed) skin (group I), those having pruritus on nondiseased (noninflamed) skin (group II), and individuals with chronic secondary lesions (group III). After assigning all patients with pruritus to one group, they are further subdivided based on pruritus etiology, including dermatological, systemic, neurological, and psychogenic pruritus. If more etiologies are evident, then the patient is considered as having mixed category of pruritus, and in those subjects where the underlying cause cannot be identified pruritus is considered as being of unknown origin [7].

According to recent studies, the point prevalence of pruritus (both acute and chronic) in the general population is estimated at about 8% to 10% based on different sources [8]. Its frequency may differ in specific groups, affecting more commonly elderly people and some specific populations, like patients on dialysis [9]. Despite the growing interest in pruritus, our knowledge about pruritus in pregnancy is quite limited and is mostly based on outdated studies [10]. As the current classification of itch has changed the approach to this symptom, we performed a cross-sectional observational study to better evaluate the prevalence and characteristics of pruritus among pregnant women.

## 2. Materials and Methods

A total of 292 consecutive pregnant women were recruited into this prospective, cross-sectional study. They were at the mean age of 30.2 ± 5.3 years and in 32.9 ± 6.4 weeks of gestation (WoG). Among the pregnant women, 184 (63.0%) were primiparas and 108 (37.0%) multiparas. About 12% of participating women had a multiple pregnancy.

The indicated parameters, age and WoG, were similar among the women with pruritus and those who did not report this symptom. All patients underwent thorough anamnesis and detailed physical examination with the special emphasis on pruritus. In addition, all women with pruritus assessed its severity according to the Visual Analogue Scale (VAS), the Verbal Rating Scale (VRS), and the 12-Item Itch Questionnaire (12-IQ). The VAS is a 10-cm long horizontal line on which the patient indicates the point corresponding to her pruritus intensity, ranging from “no pruritus” to “worst pruritus imaginable” [11]. VAS was initially used to assess the severity of pain, but it is now widely used as a tool to measure itch intensity. Finally, it was validated by our group for itch assessment in 2012 [11]. In clinical studies, it is highly recommended to use at least two methods of assessment of the intensity of pruritus [11]. Keeping this in mind, all participants also classified their pruritus with the 5-point VRS, scoring this symptom verbally as “no pruritus,” “mild pruritus,” “moderate pruritus,” “severe pruritus,” and “very severe pruritus.” All pregnant women with pruritus were asked to indicate the most severe pruritus experienced within the period of previous three days [12]. The 12-IQ consists of 12 questions about various aspects of pruritus giving the final score ranging from 0 (no pruritus) to 22 points (the most severe pruritus). In addition, the patients completed the Dermatology Life Quality Index (DLQI) to assess the quality of life impairment related to cutaneous symptoms. In order to establish the probable cause of pruritus we have followed the European Guideline on Chronic Pruritus [13]. All patients underwent basic laboratory examination and if needed, additional examination and medical consultation were performed [13]. The study was performed in accordance with the Declaration of Helsinki and was approved by Ethic Committee of Wroclaw Medical University (decision 406/2015).

### 2.1. Statistical Analysis

All results were analyzed using the software package Statistica® 12.0 (Statsoft, Krakow, Poland). Descriptive statistics included frequencies, median, and minimal and maximal values. The significance of the observed differences between groups has been determined by Mann–Whitney *U* test and *χ*^2^ test with Yates correction, if necessary. Correlations between tested parameters were verified with Spearman rank correlation test. A *p* value lower than 0.05 was considered as statistically significant.

## 3. Results

### 3.1. Prevalence of Itch

**Figure 1:**
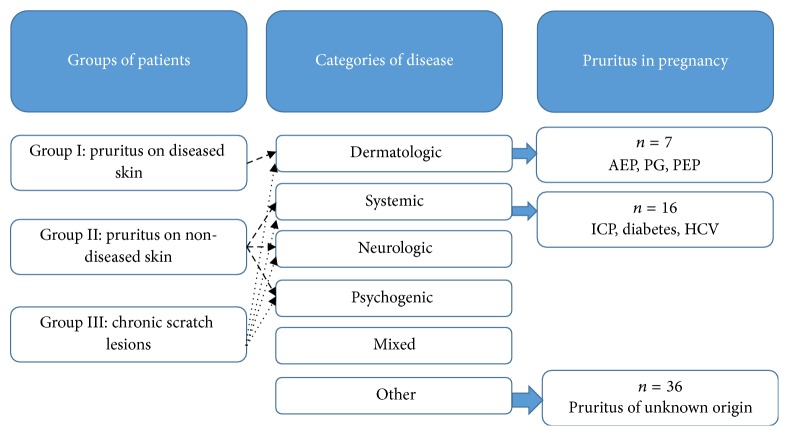
Classification on pruritus in pregnant women based on the itch classification proposed by IFSI. AEP: atopic eruption of pregnancy, PG: pemphigoid gestationis, PEP: polymorphic eruption of pregnancy, ICP: intrahepatic cholestasis of pregnancy, and HCV: chronic hepatitis C virus infection.

The prevalence of pruritus in all recruited women (entire pregnancy prevalence) was 38.0% (*n* = 111), although at the time of examination (point prevalence) it was only reported by 20.2% (*n* = 59) of patients. Twenty-two (6.7%) women experiencing pruritus suffered from this sensation before the pregnancy. Among the women with itch, 78% (*n* = 46) had a singleton gestation and 22% (*n* = 13) had a multiple pregnancy. Pruritus was more frequently connected with multiple pregnancy (multiple pregnancy: 37.1% versus singleton pregnancy: 17.9%, *p* = 0.01); however, its prevalence was unrelated to the number of previous pregnancies and number of live births. Detailed data is demonstrated in Table 1. According to current classification of itch, 7 (11.8%) out of 59 women with pruritus had dermatologic itch connected with specific dermatoses of pregnancy (AEP, PEP, and PG). The second subgroup, where systemic itch was diagnosed, consisted of 16 (27.1%) patients. In this group itch was attributed to ICP (*n* = 10), hypothyroidism (*n* = 3), gestational diabetes (*n* = 2), and chronic hepatitis C virus infection (*n* = 1), as all these diseases are known to be related to chronic itch. However, we cannot exclude the possibility that at least in some women in this group the systemic disease was not causative but just coincidental to chronic pruritus. In the remaining participants with pruritus (*n* = 36, 61.0%), the underlying cause of pruritus could not be established and it was classified as pruritus of unknown origin (Figure 1).

### 3.2. Characteristic of Pruritus

**Figure 2:**
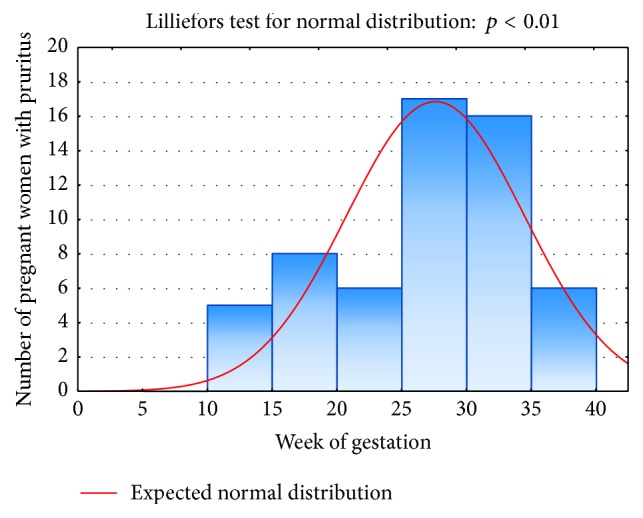
Pruritus onset depending on week of gestation.

**Figure 3:**
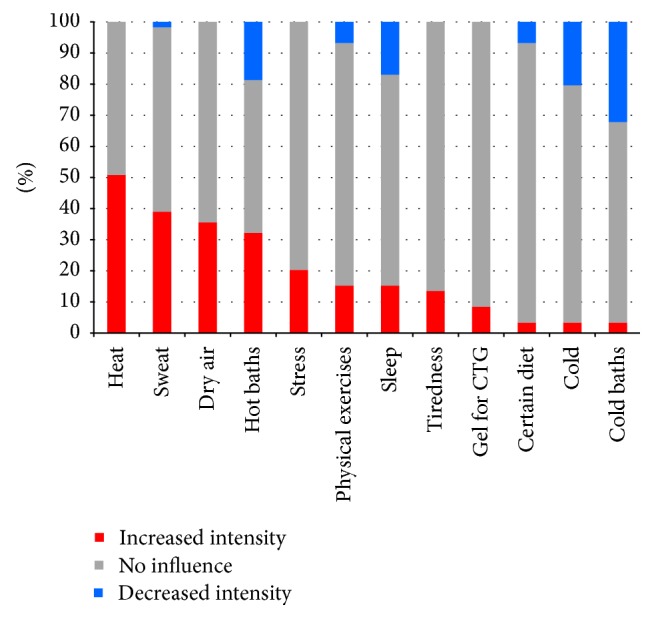
Factor exacerbating and relieving pruritus in pregnant women (CTG: cardiotocography).

Pruritus on average started at 27.2 ± 7.6 WoG. In most pregnant women, it started after the 25th WoG, although at the latest this symptom appeared at 38th WoG (Figure 2). Most commonly pruritus affected the abdomen and chest (*n* = 52 in both locations altogether, 88.1%), hands (*n* = 25, 42.4%), and feet and lower legs (*n* = 24 in each location, 40.7%) (Table 2). Surprisingly, only 3 (5.1%) women suffered from itch affecting the anogenital area. Almost one-third (32.2%) of women with pruritus presented with secondary lesions. Approximately 70% of women (69.5%) suffered from pruritus on a daily basis, whereas the remaining 30.5% reported it as appearing a few times a week. Most frequently pregnant women described itch-related sensations as tickling (52.5%, *n* = 31) and burning (44.1%, *n* = 26), followed by tingling (23.7%, *n* = 14), pinching (18.6%, *n* = 11), prickling (15.5%, *n* = 9), numbness (1.7%, *n* = 1), and pain (1,7%, *n* = 1). Moreover, the patients who suffered from pruritus reported this symptom as being predominantly annoying (59.3%, *n* = 35), burdensome (49.2%, *n* = 29), unbearable (27.1%, *n* = 16), and worrisome (15.3%, *n* = 9). Although the itch sensation appeared most frequently in the evening, more than 50% of women also reported pruritus in other times of the day or at night (Table 3). About half of pruritic participants had troubles in falling asleep (almost always: 28.8%, occasionally: 20.3%) and 42.3% (almost always: 18.6%, occasionally: 23.7%) reported awakenings because of this symptom. In addition, 3 (5.1%) pregnant women used medication for insomnia due to pruritus. Heat, dry air, and sweat were the most important factors exacerbating pruritus (Figure 3).

### 3.3. Pruritus Severity and Quality of Life Impairment

**Figure 4:**
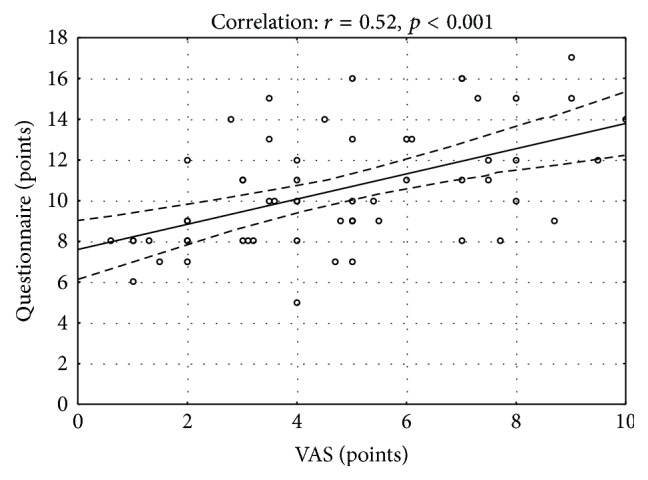
Correlation between 12-Item Itch Questionnaire scoring and VAS.

The mean intensity of pruritus measured with VAS was 4.8 ± 2.4 points ranging from 0.6 to 10 points; 8 (13.6%) described it as very mild, 17 (28.8%) as mild, 26 (44.1%) as of moderate intensity, 7 (11.9%) as severe, and 1 (1.7%) person as very severe. Regarding the 12-IQ the mean score was 10.5 ± 2.9 points (range: 5–17 which reflected 22.7% to 77.3% of the maximal itch scoring according to 12-IQ). A significant correlation between VAS and 12-IQ scores was observed (*ρ* = 0.52, *p* < 0.001) (Figure 4).

The mean DLQI scoring in patients with pruritus was 5.5 ± 5.8 points ranging from 1 to 30 points. A significant correlation was noted between DLQI scoring and pruritus intensity as assessed by the VAS (*ρ* = 0.41, *p* = 0.001) and the 12-IQ (*ρ* = 0.5, *p* < 0.001). According to DLQI 13 (22.0%) pregnant women with pruritus had normal QoL, 26 (44.1%) had slightly impaired QoL, 13 (22.0%) had moderately impaired QoL, 5 (8.5%) had severely impaired QoL, and 2 (3.4%) had extremely impaired QoL. As expected, pruritus was more frequent among women with ICP (*p* < 0.001). The higher prevalence of pruritus was also observed in women diagnosed with systemic disorders, for example, diabetes or arterial hypertension.

## 4. Discussion

Pruritus is an unpleasant sensation that provokes the desire to scratch. The itch during pregnancy may have numerous causes connected mainly with infections, infestations, particular systemic disorders (e.g., liver or kidney dysfunction), pregnancy-specific dermatoses, and exacerbation of preexisting dermatologic conditions, like atopic dermatitis [14]. This is the first study evaluating the pruritus occurring during pregnancy based on the new classification of itch as proposed in 2007 and evaluating the associated quality of life impairment connected with this symptom [7]. Pruritus gravidarum might be both localized, affecting mainly breasts and abdomen, and generalized. It may accompany the specific dermatoses of pregnancy, although it can also occur without any underlying disease. Pregnancy, a unique physiological state, brings with it endocrine and immunologic changes which may contribute to pruritus. As previously outlined, the true prevalence of pruritus among pregnant women is unknown. Our study showed that the frequency of itch during pregnancy is higher than previously suspected. Result of the study by Kenyon et al. [15] showed that the overall prevalence of itch during pregnancy was approximately 23%. According to our results, at certain periods of pregnancy, almost 40% of pregnant women may suffer from pruritus. Its occurrence seems to be most common in the third trimester. The finding is consistent with previously published observations [16, 17]. Interestingly, the majority of pregnant women in our study suffered from pruritus of unknown origin. Although all of our patients underwent detailed gynecological and dermatological examination, only 40% had an underlying cause for their pruritus. Usually the intensity of pregnancy-related pruritus was of moderate intensity. However, physicians should remember that generalized itch of greater severity (with a mean VAS = 6.6 points) commonly affecting hands and feet with deterioration during the night is frequently connected with ICP [15, 18]. Therefore, some authors classify pruritus gravidarum as with or without cholestasis [19].

The cause of itch accompanying pregnancy dermatoses is still poorly understood. Although infrequent, pregnancy dermatoses can not only cause pruritus but can also carry the risk of adverse fetal and maternal outcomes [20]. The connection between progesterone and pruritus was initially taken under consideration with regard to the pathophysiology of ICP [21]. However, recent experimental studies have suggested the role of autotaxin, and its product, lysophosphatidic acid, as possible mediators of cholestatic itch in ICP [22].

Indubitably, striae gravidarum (stretch marks) are one of the most common physiologic skin changes in pregnancy, visible in up to 90% of pregnant white women [20]. Their etiology remains unknown. Interestingly, pregnancy-associated striae may occasionally be the primary localization of PEP, a condition that typically affects primigravidas [20].

In our study, the most common location of itch, occurring in almost 90% of women reporting this symptom, was the abdomen. Similar results were observed by Kenyon et al. [15]. Abdominal pruritus in pregnancy is most related to pregnancy-induced stretching of the abdominal skin [13]. Stretching may activate dermal nerve endings leading to pruritus; however, the exact mechanism is poorly understood. In addition, damage to the collagen may induce an allergic type response contributing to the development of PEP lesions. This suggestion is supported by the fact that women with multiple pregnancies experience PEP more often.

It should be emphasized that itching appears to be a significant problem during night hours causing significant sleep disturbances in one-fifth of the pregnant women with pruritus. Some studies suggest that sleeping less than 8 hours per day during the 1st and 2nd trimester is a risk factor for miscarriage, so managing nighttime pruritus is important [23, 24].

In conclusion, pruritus during pregnancy is a complex symptom. Physicians taking care of the pregnant women affected with itch should undertake proper clinical management (for details see [13]), as it is essential for the well-being not only of the expectant mother, but also of the fetus. Additional laboratory findings and careful anamnesis with an emphasis on the location and timing of the pruritus often reveal important clues that can facilitate diagnosis and efficacious treatment. However, as many pregnant women may also suffer from pruritus of unknown origin, as in our group, further studies are needed to better characterize this subset of patients and determine the best treatment options.

## Figures and Tables

**Table 1:** Characteristic of a group of patients.

	Without pruritus	With pruritus	*p*
Age	30.2 ± 5.3	30.3 ± 5.9	0.93
Number of previous pregnancies	0.9 ± 1.3	0.9 ± 1.0	0.97
Number of previous births given	0.5 ± 1.0	0.6 ± 0.7	0.84
WoG	32.9 ± 6.4	33.1 ± 4.6	0.82
Singleton pregnancy	211 (82.1%)	46 (17.9%)	0.01
Multiple pregnancy	22 (62.9%)	13 (37.1%)	

WoG: week of gestation.

**Table 2:** Localization of pruritus.

Body area	Number of patients	Percent [%]
Abdomen	52	88.1
Chest	52	88.1
Hands	25	42.4
Shanks	24	40.7
Feet	24	40.7
Forearms	22	37.3
Thighs	21	35.6
Back	20	33.9
Shoulders and arms	19	32.2
Breasts	19	32.2
Scalp	7	11.9

**Table 3:** Occurrence of pruritus during different times of the day.

Time of the day/frequency	Not at all	Rarely	Often	All the time
Morning	9 (15.3%)	30 (50.8%)	10 (16.9%)	10 (16.9%)
Afternoon	17 (28.8%)	18 (30.5%)	17 (28.8%)	7 (11.9%)
Evening	7 (11.9%)	14 (23.7%)	21 (35.6%)	17 (28.8%)
Night	22 (37.3%)	12 (20.3%)	12 (20.3%)	12 (20.3%)
